# Systemic immune inflammation index and risk of stroke: a cross-sectional study of the National Health and Nutrition Examination Survey 2005–2018

**DOI:** 10.3389/fneur.2024.1431727

**Published:** 2024-09-12

**Authors:** Hua Xue, Yuqi Zeng, Xinyang Zou, Yongkun Li

**Affiliations:** ^1^Department of Neurology, Shengli Clinical Medical College of Fujian Medical University, Fuzhou, China; ^2^Department of Neurology, Fujian Medical University Union Hospital, Fuzhou, China

**Keywords:** NHANES, systemic immune inflammation index, stroke, logistic regression, cross-sectional study

## Abstract

**Background:**

The incidence of stroke has increased globally, resulting in medical expenditures and social burdens over the past few decades. We aimed to explore the relationship between systemic immune inflammatory index (SII) and stroke using the National Health and Nutrition Examination Survey (NHANES) from 2005 to 2018.

**Methods:**

Based on NHANES data, 902 stroke patients and 27,364 non-stroke patients were included in this study. SII was the independent variable and stroke was the dependent variable. Univariate and multivariate logistic regression analyses were used to explore the association between SII and stroke. Restricted cubic spline (RCS) method was used to test the nonlinear association between SII and stroke.

**Results:**

Weighted logistic regression analysis showed a significant association between SII and stroke (OR: 1.985, 95% CI: 1.245–3.166, *p* = 0.004). The interaction test showed that the association between SII and stroke was not significant between strata (*p* > 0.05). A significant positive association between SII and stroke risk (OR >1, *p* < 0.05) was observed in the crude model, model I and model II. RCS analysis showed no nonlinear positive association between SII and stroke risk after adjusting for all confounders.

**Conclusion:**

Our study determined that SII is associated with stroke risk. Given the inherent limitations of cross-sectional studies, further research is necessary to validate the causality of this association and to demystify the underlying mechanisms between inflammation and stroke.

## 1 Introduction

Stroke has emerged as a significant global public health concern, with a rising burden that impacts personal health, family, and societal economics (1). Despite advancements in diagnostic and therapeutic strategies for stroke over the past decades, data indicates a 2.1% increase in the global lifetime risk of stroke among adults aged 25 and above in 2016 compared to 1990 (2). Stroke encompasses ischemic stroke, which constitutes approximately 87% of cases, and hemorrhagic stroke, characterized by higher mortality rates despite its lower prevalence (3). The pathogenesis of ischemic stroke involves factors such as atherosclerotic plaque formation, cardiac emboli, thrombosis, vasospasm, and hypoperfusion (4). On the other hand, hemorrhagic stroke is often linked to weakened blood vessel walls due to high blood pressure, ruptured aneurysms, or vascular malformations (5). The underlying mechanisms of stroke are intricate, influenced by various intrinsic and extrinsic factors. Recent research underscores the significance of systemic inflammation in stroke pathogenesis, which is intertwined with both infectious and non-infectious triggers (6). In ischemic stroke, the instability of atherosclerotic plaques and subsequent vascular blockages are closely associated with inflammatory regulation (7). Similarly, the inflammatory response plays a key role in neurological damage and prognosis after hemorrhagic stroke (8). In both types of stroke, there is a robust inflammatory reaction characterized by neutrophil and macrophage activation, along with the release of inflammatory mediators like tumor necrosis factor-α (TNF-α), interleukin-6 (IL-6), and C-reactive protein (CRP) (9, 10). These processes contribute to initial damage and impact subsequent repair and regeneration (9, 10).

Systemic immune inflammation index (SII) is a novel comprehensive inflammatory index that considers three types of inflammatory immune cells (lymphocytes, neutrophils, and platelets), reflecting the balance between immunity and inflammatory status (11). Initially utilized for predicting tumor prognosis and identifying high-risk patients, SII has shown associations with disease severity and poor outcomes in various conditions, including diverse cancer, heart failure, and cardiovascular diseases (11–13). Recent studies have confirmed the relevance of SII in cerebrovascular diseases. For instance, Wang’s et al. (14) research highlighted SII as an independent risk factor for stroke-associated pneumonia in patients with intracerebral hemorrhage, correlating with unfavorable results. Furthermore, a cross-sectional study suggested a connection between SII and cerebral small vessel disease, providing evidence for the prognostic relevance of SII (15). Notably, individuals with elevated SII levels were found to be at higher risk of modified white matter hyperintensity (WMH) burden and basal ganglia enlarged perivascular spaces (BG-EPVS) (15). The study by Kelesoglu et al. (16) confirmed that the increase in serum SII is closely related to the severity of carotid artery stenosis. Specifically, neutrophils can reinfiltrate the ischemic site within the first hours after stroke and induce brain tissue damage by activating the inflammatory response through the release of inflammatory mediators (17). Lymphocytes, especially T cells and B cells, participate in the repair process after stroke by regulating immune responses. A decrease in lymphocyte counts could suggest an immunosuppressed state, impacting the recovery from stroke (18). Platelets are not only key cells for coagulation, but also participate in inflammatory reactions, releasing inflammatory mediators, promoting cerebrovascular inflammation and aggravating brain tissue damage (19, 20). Consequently, the Systemic Immune-Inflammation Index (SII), as a biomarker incorporating counts of three cellular components, provides a more nuanced reflection of the immune inflammatory landscape, enabling a finer assessment of the equilibrium between inflammatory and immune states.

While some research has indicated that SII could be a useful inflammatory markers for diagnosing and predicting stroke outcomes, there is a lack of large-scale sampling studies. The National Health and Nutrition Examination Surveys (NHANES) database, with its complex multistage probability sampling design, provides a nationally representative and ethnically diverse cohort. Our study utilized data from 7 periods of the NHANES database spanning from 2005 to 2018. We employed weighted logistic regression to develop a model that accounted for confounding factors and investigated the relationship between SII and stroke risk.

## 2 Materials and methods

### 2.1 Study design and data source

**Figure 1:**
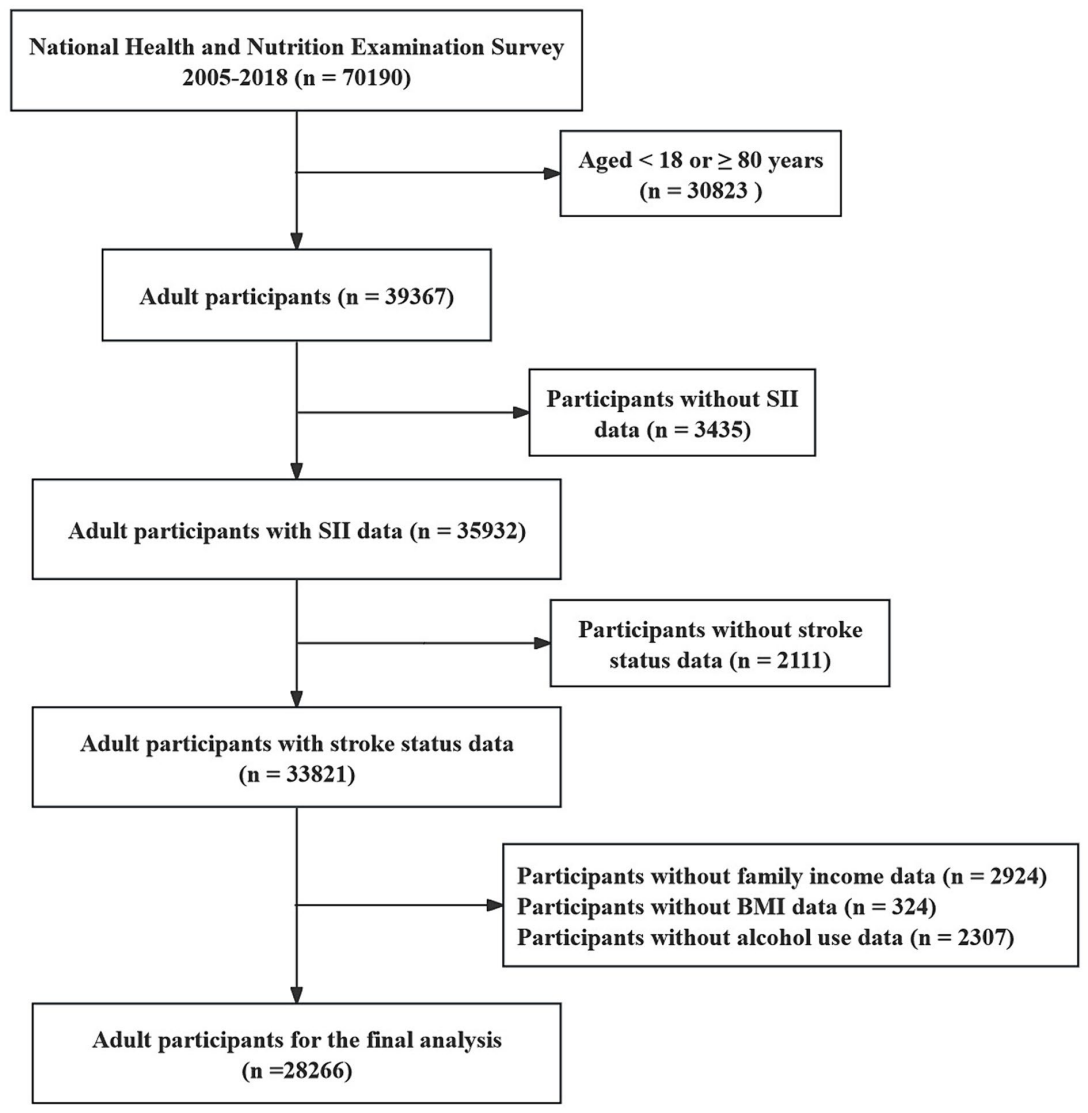
Flowchart of the participant selection from NHANES 2005–2018.

We conducted an observational cross-sectional study using data from the National Health and Nutrition Examination Survey (NHANES) to investigate the casual relationship between systemic immune inflammation indexes (SII) and the risk of stroke. NHANES is a nationally representative survey project by the U.S. Centers for Disease Control and Prevention (CDC) aimed at assessing the health and nutritional status of individuals in the United States (21). The public can download all NHANES data for free at https://www.cdc.gov/nchs/nhanes/index.htm. Our study utilized data from seven survey cycles spanning from 2005 to 2018, totaling 70,190 participants. Exclusions criteria included: (i) aged <18 or ≥80 years (*n* = 30,823); (ii) missing complete blood routine count (*n* = 3,435); (iii) missing stroke diagnosis status (*n* = 2,111); (iv) missing covariates data, such as body mass index (BMI), smoke status, alcohol use status, family income (*n* = 5,555). After screening, 28,266 participants were selected for analysis. A detailed recruitment flowchart is provided in Figure 1.

### 2.2 Definition of SII

The exposure variable in our study was SII. The SII serves as a hematologic marker used to quantitatively assess both systemic inflammation and immune status within patients (22, 23). This index is calculated by the following formula: SII = P × N/L. Herein, “P” symbolizes the platelet count, “N” denotes the neutrophil count, and “L” represents the lymphocyte count (22).

### 2.3 Stroke assessment

Stroke was defined as a previous diagnosis self-reported by a physician during a face-to-face interview. In NHANES, participants who answered the question on the medical conditions questionnaire, “Has a doctor or other health professional ever told you that you had a stroke?” where “yes” were considered to have had a stroke. In addition, although there is a lack of information on stroke type in the NHANES database, given the relatively high incidence of ischemic stroke in stroke patients, it is likely that most of the stroke participants included in this study had an ischemic stroke (24).

### 2.4 Covariates

Over the past few decades, extensive research has been conducted on the etiology and risk factors of stroke. We have endeavored to collect a comprehensive set of covariates that potentially confound the relationship with stroke, including age, gender, race/ethnicity, family income, smoke status, alcohol use, obesity, hypertension, diabetes, and coronary heart disease, all of which have been implicated in the occurrence of stroke. Race/ethnicity was categorized into five groups: non-Hispanic White, non-Hispanic Black, other Hispanic, Mexican American, and other races. Regarding smoke status, participants were defined as smokers if they answered “yes” to either the question “Have you smoked at least 100 cigarettes during your entire life?” or “Do you currently smoke?” in the questionnaire (25). With respect to alcohol consumption, participants were classified as drinkers if they responded “yes” to the question “Have you ever had at least 12 drinks of any type of alcoholic beverage in your lifetime?” in the survey (26). Hypertension was defined based on either self-reported prior diagnosis by a physician or measured blood pressure during the examination. Participants were considered hypertensive if they met at least one of the following criteria: (1) average systolic blood pressure (SBP) ≥130 mmHg; (2) average diastolic blood pressure (DBP) ≥90 mmHg; (3) self-reported history of hypertension diagnosis; or (4) current use of anti-hypertensive medication (27). Body mass index (BMI) is widely used to estimate overweight/obesity status. Clinically, BMI values greater than 25 and 30 kg/m^2^ are generally regarded as the primary diagnostic thresholds for overweight and obesity, respectively (28). Participants were deemed to have diabetes if they met at least one of the following conditions: “told by a doctor that they have diabetes,” “hemoglobin A1c (HbA1c) concentration >6.5%,” or “fasting plasma glucose (FPG) level >126 mg/dL (7.0 mmol/L)” (29). Concentrations of fasting blood glucose (FBG), hemoglobin A1c (HbA1c), red blood cell (RBC) count, neutrophil count, monocyte count, lymphocyte count, and platelet count were all determined through standardized laboratory assays.

### 2.5 Statistical analysis

Data processing and analysis in this study were performed using R statistical software and MEC weights (WTMEC2YR). The NHANES surveys utilize various intricate sampling designs, thus we incorporated sample weights for different study periods in our analytical methods to ensure precise estimates of health-related statistics (30). Continuous variables are presented as weighted means and standard deviations (SD), whereas categorical variables are presented as frequencies and percentages. To identify variances in baseline characteristics between stroke and non-stroke participants, student’s *t*-test was used for continuous variables and Chi-square test was used for categorical variables. A *p*-value <0.05 indicated statistically significant. We use the “survey” package to construct a weighted logistic regression model. Multivariable weighted logistic regression models were utilized to investigate the relationship between SII and stroke risk. To assess the correlation and potential non-linear connection between SII and stroke, the continuous SII variable was categorized into quartiles, and trend *p* was calculated. Weighted logistic regression models were constructed using the survey package, both unadjusted and adjusted for confounders, with group analyses of confounders based on significant interaction terms. Initially, an unadjusted crude model was applied, followed by two multivariable logistic regression models that progressively controlled for covariates. Model 1 adjusted for age, race, smoking, and drinking status, while Model 2 adjusted for additional factors including gender, diabetes, hypertension, and coronary heart disease. The association strength was evaluated using odds ratios (OR) and 95% confidence intervals (CI). Furthermore, the restricted cubic splines (RCS) were used to explore the non-linear relationships. To explore the threshold effect of SII on the risk of stroke is and to find the inflection point, we used the smooth curve fitting and generalized additive models.

## 3 Results

### 3.1 Baseline characteristics

Details of the baseline characteristics of all participants grouped by stroke status are provided in Table 1. A total of 28,266 participants participated in the analysis, of which 50.92% were female and 49.08% were male. The average age of the sample is 47.57 (16.44). After classifying the participants according to stroke incidence, a total of 902 participants were identified as stroke patients, accounting for 3.2% of the total sample. The results showed that there were significant differences between the stroke group and the non-stroke group in terms of age, race, family income, BMI, smoking, hypertension, diabetes, coronary heart disease, platelets, neutrophils, lymphocyte, WBC count, monocyte, glycohemoglobin and SII (*p* < 0.05). Specifically, the average age of the stroke group was 62.19 (11.92), which was significantly higher than the average age of the non-stroke group, 47.09 (16.44) (*p* < 0.001). The SII of the stroke group was 588.76 (426.56), which was significantly higher than that of the non-stroke group, 536.25 (366.50) (*p* < 0.001). In addition, there was a significant difference in glycohemoglobin between the two groups. The glycohemoglobin in the stroke group was 6.19 (1.37), which was much higher than that in the non-stroke group 5.72 (1.08) (*p* < 0.001).

**Table 1:** Characteristics of NHANES participants between 2005 and 2018.

Characteristic	Total	Non-stroke	Stroke	*p*-value
Overall	28,266	27,364	902	
Sex, *N* (%)				0.87
Female	14,393 (50.92)	13,936 (59.93)	457 (50.66)	
Male	13,873 (49.08)	13,428 (49.07)	445 (49.33)	
Age, (y), mean (SD)	47.57 (16.44)	47.09 (16.44)	62.19 (11.92)	<0.001
Race, *N* (%)				<0.001
Mexican American	4,512 (15.96)	4,414 (16.13)	98 (10.86)	
Other Hispanic	2,656 (9.39)	2,600 (9.50)	56 (6.21)	
Non-Hispanic White	12,038 (42.59)	11,640 (42.54)	398 (44.12)	
Non-Hispanic Black	6,000 (21.23)	5,714 (20.88)	286 (31.71)	
Other race	3,060 (10.83)	2,996 (10.95)	64 (7.10)	
BMI, (kg/m^2^), *N* (%)				<0.001
<25	7,913 (28.03)	7,716 (28.19)	197 (21.84)	
25–30	9,194 (32.53)	8,931 (32.64)	263 (29.16)	
≥30	11,159 (39.48)	10,717 (39.16)	442 (49.00)	
PIR, *n* (%)				<0.001
<1.3	8,804 (31.15)	8,409 (30.73)	395 (43.79)	
1.3–3.5	10,511 (37.19)	10,152 (37.10)	359 (39.80)	
>3.5	8,951 (31.67)	8,803 (32.17)	148 (16.41)	
Smoke, *N* (%)				<0.001
No	15,453 (54.67)	15,130 (55.29)	323 (35.81)	
Yes	12,813 (45.33)	12,234 (44.71)	579 (64.19)	
Alcohol use, *N* (%)				0.09
No	7,048 (24.93)	6,802 (24.86)	246 (27.27)	
Yes	21,218 (75.06)	20,562 (75.14)	656 (72.72)	
Hypertension, *N* (%)				<0.001
No	18,603 (65.81)	18,389 (67.20)	214 (23.72)	
Yes	9,663 (34.18)	8,975 (32.80)	688 (76.28)	
Diabetes, *N* (%)				<0.001
No	24,743 (87.54)	24,160 (88.29)	583 (64.63)	
Yes	3,523 (12.46)	3,204 (11.71)	319 (35.37)	
CHD, *N* (%)				<0.001
No	27,319 (96.64)	26,566 (97.08)	753 (83.48)	
Yes	9,38 (3.36)	798 (2.92)	149 (16.52)	
Platelets, (10^9^ cells/L), mean (SD)	249.36 (66.04)	249.56 (65.61)	243.19 (77.77)	0.015
Neutrophils, (10^9^ cells/L), mean (SD)	4.29 (1.80)	4.28 (1.80)	4.52 (1.78)	<0.001
Lymphocyte, (10^9^ cells/L), mean (SD)	2.19 (2.31)	2.19 (2.34)	2.11 (0.79)	0.007
WBC, (10^9^ cells/L), mean (SD)	7.28 (3.28)	7.27 (3.30)	7.49 (2.28)	0.006
Monocyte, (10^9^ cells/L), mean (SD)	0.55 (0.20)	0.55 (0.20)	0.59 (0.23)	<0.001
Glycohemoglobin, (%), mean (SD)	5.74 (1.09)	5.72 (1.08)	6.19 (1.37)	<0.001
SII, mean (SD)	537.93 (368.68)	536.25 (366.50)	588.76 (426.56)	<0.001

Continuous and categorical variables are expressed as mean (SD) and *n* (%), respectively. Mean (SD) and *n* (%) have been weighted. BMI, body mass index; PIR, income to poverty ratio; CHD, coronary atherosclerotic heart disease; WBC, white blood cell; SII, systemic immune inflammation index.

### 3.2 Univariate logistic regression analysis of stroke

After performing a weighted univariate logistic regression analysis (Table 2), our results indicate that older age (≥ 60 years), female, non-Hispanic White, non-Hispanic Black, other race, high BMI (>25), smoking, hypertension (yes), diabetes (yes) were at increased risk of stroke (OR >1, *p* < 0.05). However, participants who were other Hispanic, PIR (<1.3) show a reduced risk of stroke (OR <1, *p* < 0.05).

**Table 2:** Weighted univariate logistic analysis of stroke.

Characteristic	OR 95% CI	*p*-value
Age		
<60	ref	ref
≥60	5.306 (4.486, 6.267)	<0.001
Sex		
Male	ref	ref
Female	1.206 (1.011, 1.439)	0.037
Race		
Mexican American	ref	ref
Other Hispanic	0.904 (0.616, 1.326)	0.603
Non-Hispanic White	1.573 (1.232, 2.008)	<0.001
Non-Hispanic Black	2.553 (1.232, 2.008)	<0.001
Other race	1.799 (1.172, 2.762)	0.007
BMI, (kg/m^2^)		
<25	ref	ref
25–30	1.130 (0.879, 1.425)	0.334
≥30	1.704 (1.332, 2.182)	<0.001
PIR		
<1.3	ref	ref
1.3–3.5	0.690 (0.557, 0.856)	<0.001
>3.5	0.301 (0.236, 0.386)	<0.001
Smoke		
No	ref	ref
Yes	2.105 (1.785, 2.482)	<0.001
Alcohol use		
No	ref	ref
Yes	1.206 (1.109, 1.149)	0.008
Hypertension		
No	ref	ref
Yes	6.383 (5.282, 7.714)	<0.001
Diabetes		
No	ref	ref
Yes	4.983 (4.088, 6.073)	<0.001

### 3.3 Relationship between stroke and SII

After performing a weighted multivariate logistic regression analysis (Table 3), our results indicate that a higher SII score is associated with an increased risk of developing stroke. This association was significant in our crude model (OR = 1.985; 95% CI = 1.245–3.166, *p* = 0.004) and model 1 (OR = 1.728; 95% CI = 1.118–2.672, *p* = 0.014). In the fully adjusted model, the positive association between SII and stroke remained stable (OR = 1.562; 95% CI = 1.020–2.394, *p* = 0.040), indicating that for every unit increase in log-formed SII, the risk of developing stroke increased by 16%. We further transformed the SII from a continuous variable into a categorical variable (quartiles) for sensitivity analysis (Table 3). Compared with the lowest quartile, the risk of developing stroke in the highest quartile increased by 48% (OR = 1.481; 95% CI = 1.121–1.958, *p* = 0.006) in the crude model, 39% (OR = 1.394; 95% CI = 1.057–1.838, *p* = 0.018) in the model 1 and 34% (OR = 1.348; 95% CI = 1.010–1.800, *p* = 0.042) in the model 2. In addition, there was a significant trend in SII and stroke risk with quartile (*p* for trend <0.05).

**Table 3:** Weighted multivariate logistic analysis log-formed SII and stroke.

	Crude model		Model 1		Model 2	
	OR 95% CI	*p*-value		*p*-value		*p*-value
Log-formed SII	1.985 (1.245, 3.166)	0.004	1.728 (1.118, 2.672)	0.014	1.562 (1.020, 2.394)	0.040
Stratified by log-formed SII quartiles						
Q1	ref		ref			
Q2	1.116 (0.845, 1.472)	0.433	1.095 (0.829, 1.447)	0.515	1.189 (0.893, 1.583)	0.231
Q3	1.053 (0.763, 1.451)	0.750	1.021 (0.734, 1.419)	0.898	1.064 (0.767, 1.474)	0.706
Q4	1.481 (1.121, 1.958)	0.006	1.394 (1.057, 1.838)	0.018	1.348 (1.010, 1.800)	0.042
*p* for trend		0.011		0.033		0.080

### 3.4 Subgroup analysis and interaction test

We also conducted stratified analyses to investigate whether the association between SII and stroke incidence remained consistent across different subgroups (Table 4). Our subgroup analyses revealed that the positive correlation was not significantly altered by stratification variables including gender (males and females), age (<60, ≥60), BMI (<25, 25–30, ≥30), PIR (<1.3, 1.3–3.5, >3.5), smoking status, alcohol use, diabetes, and hypertension. There was no statistically significant difference in the relationship between SII and stroke across these strata, as indicated by the interaction test *p*-values >0.05, suggesting that covariates have no significant effect on this association.

**Table 4:** Subgroup analysis for the association between SII and stroke.

Characteristic	OR 95% CI	*p*-value	*p* for interaction
Age			0.197
<60	2.605 (1.296, 5.234)	<0.001	
≥60	1.523 (0.917, 2.530)	0.103	
Sex			0.699
Male	2.111 (1.141, 3.906)	0.017	
Female	1.775 (0.977, 3.223)	0.059	
BMI, (kg/m^2^)			0.260
<25	2.434 (1.502, 5,580)	0.003	
25–30	1.543 (0.559, 4.260)	0.401	
≥30	1.463 (0.858, 2.495)	0.162	
PIR			0.056
<1.3	3.121 (1.885, 5.165)	<0.001	
1.3–3.5	2.348 (1.181, 4.669)	0.014	
>3.5	0.603 (0.226, 1.607)	0.312	
Smoke			0.968
No	1.791 (0.905, 3.542)	0.093	
Yes	1.840 (1.073, 3.155)	0.026	
Alcohol use			0.916
No	2.057 (0.961, 4.404)	0.063	
Yes	1.956 (1.175, 3.256)	0.009	
Hypertension			0.219
No	1.019 (0.476, 2.179)	0.960	
Yes	1.911 (1.178, 3.101)	0.008	
Diabetes			0.218
No	2.063 (1.168, 3.645)	0.012	
Yes	1.219 (0.684, 2.172)	0.501	

### 3.5 The nonlinear relationship between stroke and SII

**Figure 2:**
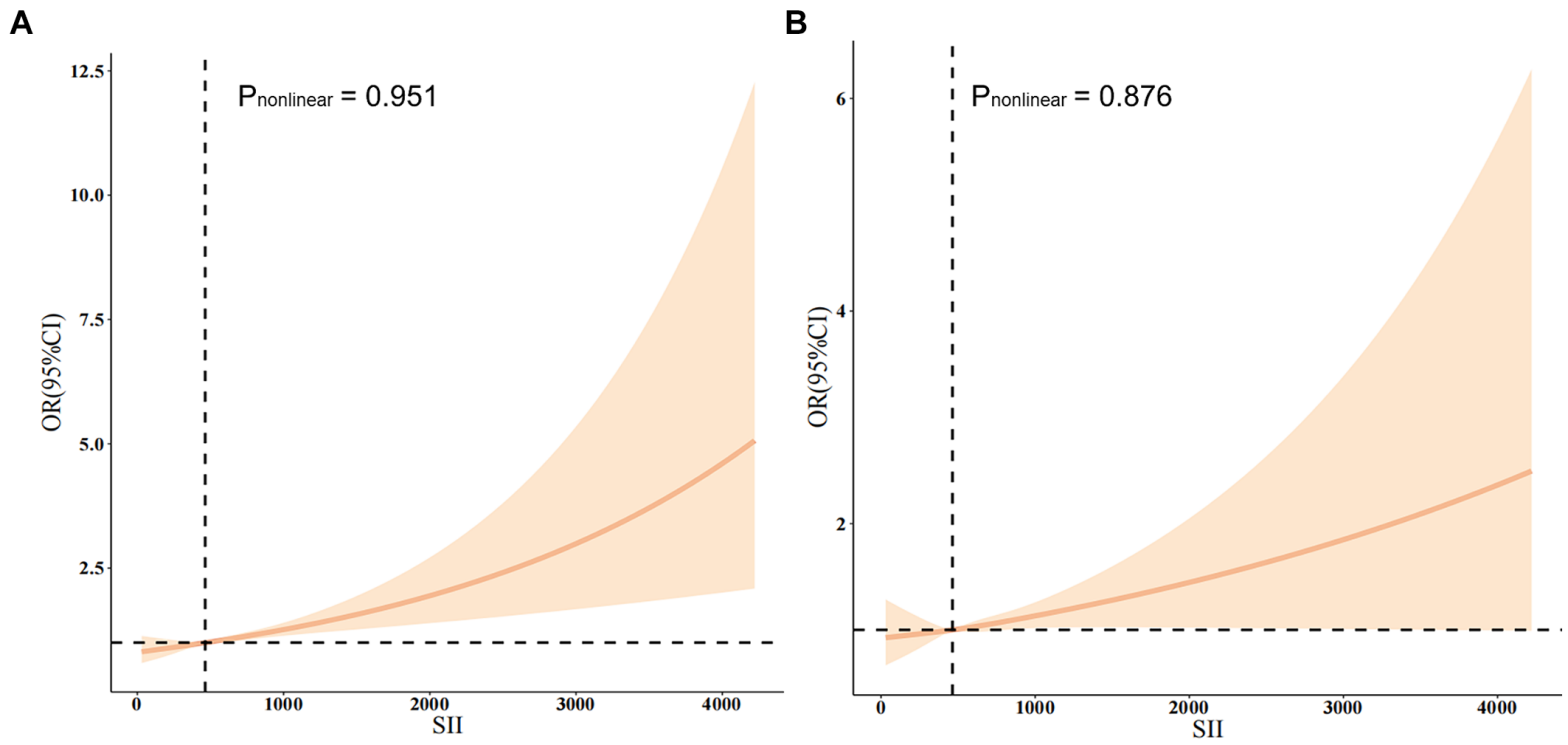
**(A)** The non-adjusted relationship between SII and stroke. **(B)** The full-adjusted relationship between SII and stroke.

In this study, restricted cubic splines (RCS) were employed to elucidate the nonlinear association between the SII and the risk of stroke (Figure 2). Covariates adjusted for in the analysis comprised gender, age, PIR, BMI, diabetes, hypertension, smoking status, and alcohol use. There was no statistically significant nonlinear relationship between SII and stroke risk (*p* > 0.05) either in the crude model (Figure 2A) or after adjustment for multiple confounders (Figure 2B). Of note, a threshold effect emerged, with a turning point observed at an SII value of 464.47. Below this critical threshold, the incidence risk of stroke remained relatively stable or even decreased; conversely, surpassing this threshold led to a marked escalation in the risk of stroke.

## 4 Discussion

This study ultimately included 28,266 participants from the NHANES 2005–2018 cohort for analysis, including 13,873 men and 14,393 women. Among them, 902 patients suffered from stroke. In baseline data, stroke patients had higher SII levels compared with normal subjects. The results of weighted univariate logistic analysis showed that there was a significant effect on the incidence of stroke among those age ≥60, BMI ≥30, diabetic, hypertensive, drinkers, and smokers. Furthermore, after adjusting for all covariates, we found that the relationship between SII levels and stroke was no nonlinear. When the SII was higher than 464.47, the risk of stroke increased significantly. Multiple imputation sensitivity analysis confirmed the association between SII and stroke.

Many epidemiologic studies have shown that the inflammatory response is associated with the stroke. Peripheral blood monocyte to lymphocyte ratio (MLR), neutrophil-to-lymphocyte ratio (NLR), platelet-to-lymphocyte ratio (PLR), and SII, as emerging biomarkers of inflammation, have been associated with prognosis of stroke patients (18, 31–35). In a cross-sectional study involving the participation of 4,854 patients at high risk for acute nondisabling cerebrovascular events, patients with minor strokes were divided into 4 groups based on quartiles of neutrophil count or neutrophil ratio, 495 of whom had a recurrent stroke after 90 days of follow-up. The study demonstrated that high levels of neutrophil count and neutrophil ratio were associated with an increased risk of new stroke, composite events, and ischemic stroke in patients with minor ischemic stroke (31). Additionally, in a study involving 796 patients with acute ischemic stroke who underwent endovascular thrombectomy, higher NLR and PLR were significantly associated with adverse outcomes (33). Ordinarily, an upsurge in platelet and neutrophil counts correlates with heightened inflammatory processes, whereas a decline in lymphocyte levels can be indicative of immune suppression or exhaustion. As an inflammatory marker containing three cell component counts, a significant elevated SII value may signal a profound inflammatory reaction or a state of immune dysregulation (23). This versatile biomarker has found broad applications across various domains of clinical research. It plays a crucial role in estimating the prognosis of diseases, evaluating disease activity levels, and tracking therapeutic efficacy. Hu et al. (35) studied the relationship between in-hospital mortality and SII in 463 stroke patients. The results showed that in-hospital mortality was positively correlated with SII, but not linearly correlated. High SII was associated with poor prognosis in acute ischemic stroke (AIS) patients.

In ischemic stroke, damaged brain cells produce large amounts of inflammatory cytokines, chemokines, reactive oxygen species (ROS), and other neurotoxic substances, which mediate blood-brain barrier disruption and inflammatory cascade reactions, while directing immune inflammatory cells into brain tissue, further mediating secondary neuronal damage and aggravating neurological dysfunction (36). Neutrophils are the initial cells to infiltrate the ischemic brain tissue. Upon arrival at the ischemic site, they release pro-inflammatory mediators, proteases, ROS, and extracellular matrix metalloproteinase (MMP), leading to secondary damage in the ischemic brain cell (37). Monocytes are capable of secreting MMP-9, an enzyme that is involved in extracellular matrix (ECM) remodeling *in vivo* and is important for tissue repair, inflammatory response, angiogenesis, and disease progression (38). MMP-9 can infiltrate into infarcted foci and exacerbate brain damage (38). Increased monocyte count has been shown to be an independent predictor of poor stroke prognosis (39). Platelets interact directly with circulating leukocytes to form platelet-leukocyte aggregates through the alteration of P-selectin and CD40 expression on the cell surface (40). The release of fibrinogen, fibronectin, platelet factor-4, and other mediators from α-granules in platelets contributes to platelet adhesion, aggregation, and the coagulation process, potentially exacerbating thrombosis (41). Conversely, lymphocytes are believed to play a crucial role in inflammation-induced neuroprotection and serve as the primary immunomodulators for brain protection following ischemic stroke (42). The role of lymphocytes in AIS varies depending on the subtype. CD4^+^ and CD8^+^ T cells can exacerbate inflammatory reactions and contribute to neuronal death by producing inflammatory factors like interferon γ and interleukin-17 (IL-17) (43). On the other hand, regulatory T cells release IL-10 through various signaling pathways, such as signal transduction, phosphatidylinositol and transcription activator pathways, phosphatidylinositol-3-kinase pathway, and mitogen-activated protein kinase pathway, which have neuroprotective effects (44).

In the context of secondary brain injury following hemorrhagic stroke (45), immune inflammation can exacerbate cerebral edema, enlarge hematoma, raise intracranial pressure, and progressively deteriorate neurological function (45). This response involves the release of various inflammatory factors, including IL-6, IL-1β, and C-reactive protein (CRP) among others (46). These inflammatory factors can attract immune cells such as neutrophils, monocytes, and macrophages to accumulate in the affected area, intensifying local inflammation and tissue damage (47). Additionally, excessive oxidative stress and excitotoxicity can lead to nerve cell death and worsen brain injury (48). Luo et al. (49) conducted a study using the SII to forecast the prognosis of subarachnoid hemorrhage (SAH). The area under the curve (AUC) of SII in predicting poor prognosis was 0.692, indicating that SII could serve as a novel independent prognostic indicator for SAH patients in the initial stages of the condition.

In the clinical management of stroke patients, early detection and risk stratification can be conducted based on the SII level, allowing for personalized management and treatment according to the inflammatory response severity. Monitoring SII levels can also help evaluate intervention effectiveness and detect potential recurrence or exacerbation early during long-term follow-up, enabling timely adjustments in treatment options. The correlation between SII and stroke has significant implications for clinical guidance in screening, etiology research, treatment, prognosis evaluation, and also provides more targeted clinical treatment.

This study, based on the nationally representative NHANES database, analyzed 28,266 participants from the United States to determine that SII levels are positively associated with stroke risk. The use of a weighted logistic regression model in the analysis, adjusting for covariates, enhanced the accuracy and reliability of the conclusions. Additionally, the large sample size and subgroup analysis contributed to the reliability and representativeness of study. The study highlights the potential value of measuring systemic inflammatory biomarkers in identifying individuals at risk for stroke, offering new options for stroke diagnosis and treatment. However, this study has several limitations. First, the cross-sectional design employed made it challenging to establish a causal relationship between exposure factors and outcome variables. Future research should prioritize prospective studies to elucidate the causal link between SII and stroke to a deeper understanding of the association. Second, although we adjusted for covariates, residual confounding factors cannot be ruled out. Notable examples include hypercholesterolemia, physical activity status, and family history of stroke. Third, due to limitations in the database, we were unable to categorize the survey questions regarding alcohol consumption. Fourth, Q2 and Q3 are not related in all models. In the fully adjusted model, SII was associated with stroke in its highest quartile. It can be seen that SII can be used as a predictor of stroke only when SII is at the highest level. Since some typical inflammatory factors (such as TNF-α, IL-6, IL-10, etc.) are not recorded in NHANES, relevant indicators cannot be included to obtain more comprehensive results. Lastly, understanding the association between SII and various stroke subtypes (e.g., large-artery atherosclerotic stroke, cardiogenic stroke) is also constrained by limitations within the NHANES database.

## 5 Conclusion

This cross-sectional study based on the NHANES database demonstrated that SII is associated with stroke risk. Given the inherent limitations of cross-sectional studies, further research is necessary to validate the causality of this association and to demystify the underlying mechanisms between inflammation and stroke.

## Data Availability

Publicly available datasets were analyzed in this study. This data can be found here: https://www.cdc.gov/nchs/nhanes/ index.htm.
